# Variations in the Fecal Microbiota of Red Deer in Relation to the Hunting Area in the Friuli-Venezia Giulia Region, Italy

**DOI:** 10.3390/ani15172517

**Published:** 2025-08-27

**Authors:** Bruno Stefanon, Valentina Cecchini, Sandy Sgorlon, Monica Colitti

**Affiliations:** Department of Agrifood, Environmental and Animal Science, University of Udine, 33100 Udine, Italy; bruno.stefanon@uniud.it (B.S.); valentina.cecchini@uniud.it (V.C.); sandy.sgorlon@uniud.it (S.S.)

**Keywords:** red deer, microbiota, hunting reserve, habitat

## Abstract

The gut microbiota of red deer is a topic of growing interest, particularly with regard to its ecological and physiological importance. The diversity and composition of these microbial communities is influenced by environmental variables, diet and interactions with other organisms in their habitat. Fecal samples were collected in 10 hunting reserves in the Friuli-Venezia Giulia region, Italy. In each hunting reserve, five individual samples were taken from the ground for a total of 50 samples. After DNA extraction, the V3–V4 16s rRNA regions were sequenced. The raw data were uploaded to QIIME2 and taxonomic annotation was performed. Significant differences between the deer sampled in the hunting reserves (*p* < 0.05) were calculated for the phyla Firmicutes and Cyanobacteriota and for the ratio of Firmicutes to Bacteroidota, especially for the mountain reserves in the north and northeast compared to the reserves in the hills in the west. Shannon and Chao1 alpha diversity indices varied between reserves (*p* < 0.01) and the Bray–Curtis beta diversity index also indicated differences in microbiota between hunting reserves. The different habitats and vegetation of the reserves could explain the differences observed. Understanding the dynamics of the microbiota of red deer provides important information about their health and feeding behavior and also has far-reaching implications for species conservation.

## Simple Summary

The gut microbiota of red deer plays a crucial role in their ecological interactions and physiological functions. This study analyzed microbiota of fecal samples from ten hunting reserves in Friuli-Venezia Giulia, Italy, using 16S rRNA sequencing of the V3–V4 regions. Taxonomic annotation revealed significant differences in the abundance of Firmicutes and Cyanobacteriota and the Firmicutes to Bacteroidota ratio, with variation most pronounced between mountainous and hilly reserves. Diversity indices, including Shannon, Chao1, and Bray–Curtis, demonstrated notable variability in microbial composition across the sites. These findings suggest that habitat type and vegetation influence gut microbiota diversity, offering insights into red deer health, feeding behavior, and broader implications for species conservation.

## 1. Introduction

The microbiota is the largest and most complex microecosystem in animals. It is influenced by the dietary habits and environment of the host, and its composition and diversity play an important role in nutrient metabolism, immunity and adaptation to the environment [1,2,3]. The gut microbiota of cervids is a topic of growing interest in animal biology, especially with regard to its ecological and physiological importance [4]. The microbiota plays a key role in digestion, nutrient fermentation, and the assimilation of essential biomolecules. The diversity and composition of these microbial communities is influenced by environmental variables, diet and genetic factors, but also by seasonal changes and interactions with other organisms in their habitat [5,6]. Understanding the dynamics of the microbiota of cervids not only provides us with important information about their health and feeding behavior but also has far-reaching implications for species conservation [7,8].

In a recent paper, it was shown that leaf-associated microbial communities are subject to seasonal fluctuations influenced by environmental stimuli and disturbances [9]. This may be of particular interest in herbivores, as different plants harbor different microbial communities that may influence the composition of the gut microbiota when these plants are consumed [10]. For example, plants with a high fiber content can promote the growth of fiber-degrading bacteria in the gut [11]. The microbiome plays a crucial role in the health of the host. It influences energy metabolism and the production of important metabolites, including short-chain fatty acids (SCFAs), which are essential for gut health and can also influence systemic physiological processes [12]. The most important SCAFAs are acetate, propionate and butyrate, which contribute a good percentage to the energy requirements of animals [13].

The metabolism of the intestinal microbiota is closely linked to the dietary habits of the host and adapts the content of digestive enzymes to the dietary habits over time. A decrease in fecal microbial diversity can reduce the functional efficiency of the microbiota and its resistance to pathogens, as has been observed also in humans [14]. Indeed, studies have shown that nutritional stress, such as in winter when food is scarce, can alter the abundance of certain gut microbes, which may affect the deer’s ability to digest food and absorb nutrients [5].

Interestingly, red deer in captivity had a higher alpha diversity of gut microbiota than their wild counterparts [1]. This homogenization of the microbiome could be due to limited foraging diversity and increased interaction between animals under restricted or temporarily restricted conditions.

The first aim of this study was to describe the composition of the fecal microbiota of red deer in a small region of north easter of Italy, Friuli-Venezia Giulia. Despite its small size, this region has a high biological, ecological and landscape richness. This great heterogeneity of environmental factors is enriched by its transitional biogeographical position between the Illyrian, Alpine and Mediterranean worlds [15]. The regional territory has 100 different types of habitats distributed in seven main natural landscapes: Alps; Prealps; Hills; Karst and Triestina Coast; Upper Plain; Lower Plain; and Lagoon [15]. Among these different habitats, there are ten hunting reserves where the red deer population exceeds 3 individuals/km^2^. The second objective was to investigate the relationship, if any, between habitats in hunting reserves and the fecal microbiota of red deer.

## 2. Materials and Methods

### 2.1. Study Area Description

**Figure 1:**
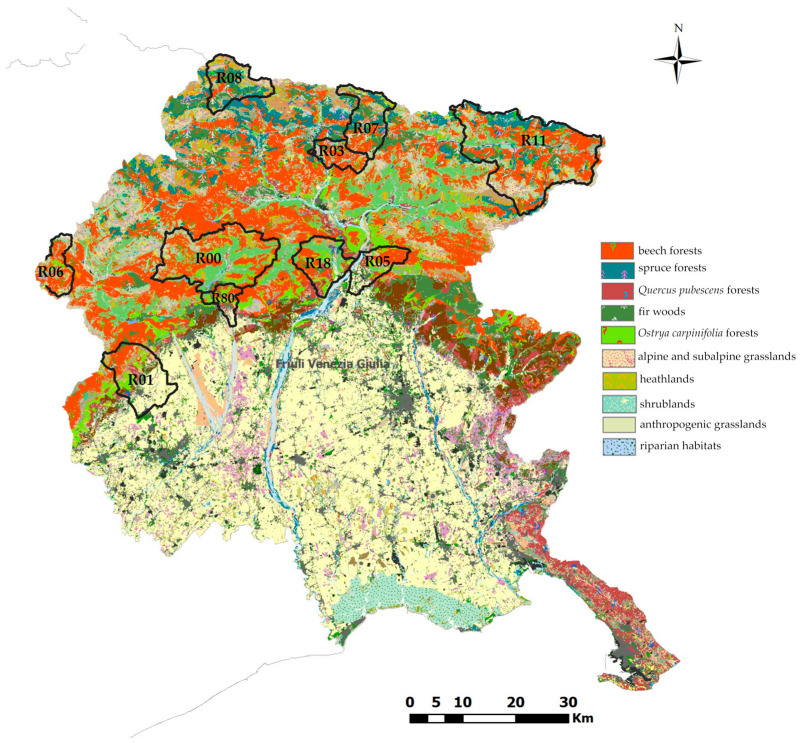
Nature map of Friuli-Venezia Giulia and hunting reserves where fecal samples were collected [15].

This study was conducted in the Friuli-Venezia Giulia region (FVG) in north-eastern Italy. Fecal samples were searched and collected in 10 hunting reserves, designated R00, R01, R03, R05, R06, R07, R08, R11, R18 and R80, in hilly and mountainous areas (Figure 1). Table 1 describes the main habitats and vegetation of the selected reserves based on the nature map of Friuli-Venezia Giulia [15]. The fecal samples were collected in 2024 in the months of April and May, which coincides with the end of the red deer congregation.

### 2.2. Sampling Procedure

In each hunting reserve, a collaboration with the hunters was agreed and the project was presented in a preliminary meeting. The hunters were instructed to collect the feces from the ground to avoid contamination with the soil and not to collect the feces in the same place and on the same day in order to avoid re-sampling the same deer. The hunters were given a collection kit with sterile gloves and a paper form on which the day of collection and the coordinates of the collection site were noted. Two drops were taken from each pile and placed in a collection tube containing 4 mL of Shield (Zymo Research, Irvine, CA, USA). This shield inactivates pathogens and preserves the genetic integrity of the DNA/RNA at room temperature for at least 20 days for subsequent analysis. During sampling, hunters searched for feces in deer-frequented hunting reserves and 5 samples were collected from 10 hunting reserves. The fecal samples from each hunting reserve were collected on the same day. To reduce the likelihood that the samples came from the same individual, only one sample was taken from the central part of the visible fresh pile, with a minimum distance of more than 2 m between each pile [13]. Dryness, texture, color and firmness were the parameters that indicated the freshness of the feces. The sex and age of the deer were not taken into account as the process of defecation was not observed. Samples were delivered to the laboratory of Department of AgroFood, Environmental and Animal Science within 20 days from collection for microbiome analysis and then stored at −20 °C until analysis.

### 2.3. DNA Extraction and Sequencing of 16S rRNA Gene Amplicons

Approximately 150 mg of each fecal sample was used for DNA extraction using Quick DNA Fecal/Soil Microbe (Zymo Research, Irvine, CA, USA), including a preparatory bead-beating step. DNA concentration was measured using a QubitTM Fluorometer (Thermo Scientific; Waltham, MA, USA). The V3 and V4 regions of the 16S rRNA were amplified by PCR for library preparation, using long primers ([16]; 16S_Amplicon_PCR_Fw: TCGTCGGCAGCGTCAGATGTGTATAAGAGACAGCCTACGGGNGCWGCAG; 16S_Amplicon_PCR_Rv: GTCTCGTGGGCTCGGAGATGTGTATAAGAGACAGGACTACHVGGGTATCTAATCC) containing the Illumina adaptor sequences. After amplification, AMPure XP bead clean up (A63880l; Beckman; Indianapolis, IN, USA) was used for purification. To attach dual-index and Illumina sequencing adapters (Nextera XT Index Kit, Illumina; San Diego, CA, USA), a second PCR was performed, followed by AMPure XP bead clean up. The Bioanalyzer (Agilent, Santa Clara, CA, USA) was used to verify the size, integrity and purity of the amplicons. The concentration of the library was measured using the QubitTM Fluorometer (Invitrogen, Carlsbad, CA, USA). Libraries were pooled at equimolar concentrations and sequenced to 2 × 250 bp paired-end using the Novaseq platform (Illumina; San Diego, CA, USA). The raw data were uploaded to the NCBI Sequence Read Archive (BioProject PRJNA1291720).

### 2.4. Bioinformatic and Statistical Analysis

Raw data in FASTQ format were uploaded to a remote server and analyzed using Qiime2-amplicon-2024.5 [17] with the DADA2 command, which includes quality filtering, ASV denoising and chimera removal. The representative sequences were aligned with mafft. A total of 39,328,490 reads were annotated, which corresponds to an average number of 786,569 per sample (maximum number per sample 1,938,690 and minimum number per sample 437,950). For taxonomic annotation, the 2024.09 backbone.full-length.nb (https://ftp.microbio.me/greengenes_release/current/ (accessed on 21 August 2025)) greengene classifier was used, which corresponds to the classification of the International Code of Nomenclature of Prokaryotes (ICNP, https://www.the-icsp.org/index.php/code-of-nomenclatur (accessed on 13 August 2025)). Taxa were assigned to genus level and uploaded to Microbiome Analyst (https://www.microbiomeanalyst.ca/ (accessed on 21 August 2025)) for statistical analysis of the microbiome [18], with counts normalized to the total (relative abundance, RA).

The Shannon and Chao1 alpha diversity indices between hunting reserves were compared with the Kruskal–Wallis test and with the FDR multiple test (*p* < 0.05) for pairwise comparison, using Prism 9 software. The comparison of beta diversity between hunting reserves was evaluated using the Bray–Curtis method. Permutational multivariate analysis of variance (PERMANOVA) was applied to assess differences in community composition between hunting reserves, and results were visualized in principal coordinate analysis (PCoA). Linear discriminant analysis (LDA) effect size (LEfSe) was then applied with a statistical threshold of *p* < 0.01 after FDR correction [19]. The estimated effect size of significant features was standardized for mean and standard deviation and uploaded to Heatmapper (http://www.heatmapper.ca (accessed on 8 July 2025)) for cluster analysis using average linkage and Spearman rank correlation.

## 3. Results

**Figure 2:**
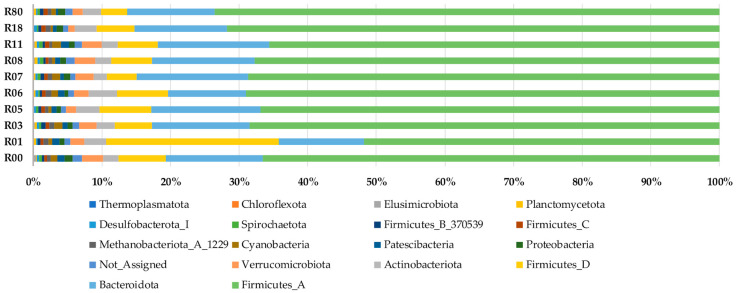
Relative abundance of fecal microbiota of red deer at phylum taxonomic level among the 10 hunting reserves.

The composition of the fecal microbiota of red deer showed a predominant RA of the phyla Firmicutes (A–D), Firmicutes_A, Bacteroidota and Firmicutes_D, with a mean percentage and standard deviation of 75.2 ± 4.6, 67.1 ± 8.2, 14.2 ± 3.1 and 7.1 ± 8.6, respectively (Figure 2). Only the phyla Verrucomicrobiota and Actinobacteriota had a mean percentage greater than 1% (3.0 ± 2.7 and 2.2 ± 1.3, respectively), while the others accounted for less than 1%.

**Figure 3:**
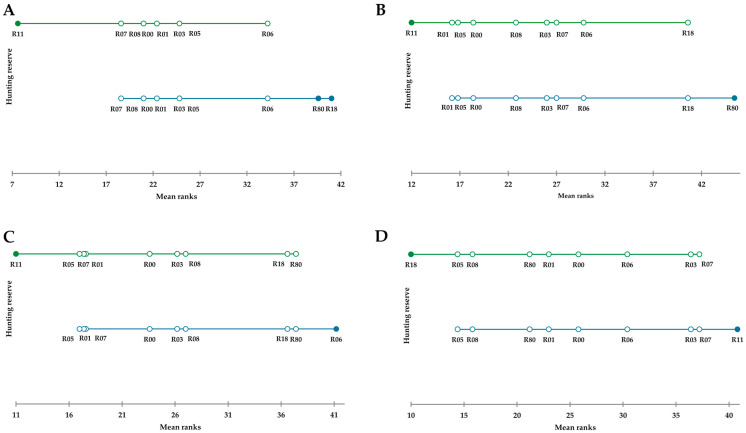
Demsar plots showing the average ranking of red deer microbiota samples at the phylum taxonomic level across the 10 hunting reserves. (**A**) Firmicutes (A–D). (**B**) Firmicutes_A. (**C**) Ratio of Firmicutes_A to Bacteroidetes. (**D**) Cyanobacteria. Lines with different colors indicate hunting reserves clustered together according to the mean rank values. Full dots indicate hunting reserves which significantly differed for *p* < 0.05.

Firmicutes (A–D) had the highest rank (*p* = 0.004) in reserves R80 and R18 and the lowest in reserve R11 (Figure 3A). Firmicutes_A, mainly representing the class Clostridia, differed significantly (*p* = 0.003) between reserves, and the highest rank was observed in reserve R80 and the lowest in reserve R11, with very close ranks for R01, R05, and R00 (Figure 3B). The ratio of Firmicutes_A to Bacteroidota varied between reserves (*p* = 0.009) and was lowest in reserve R11 and highest in reserve R06 (Figure 3C), being equal to 4.05 + 0.13 and 6.75 + 1.90, respectively. The hunting reserves R05, R01 and R07 had similar rank values (mean values of the ratios of 4.15 + 0.54, 4.19 + 0,56 and 4.28 + 0.41, respectively). There were also differences in cyanobacteria between the reserves (*p* = 0.005). They were higher in the hunting reserve R11 and lower in R18 (Figure 3D), although the mean RA was less than 1.

**Figure 4:**
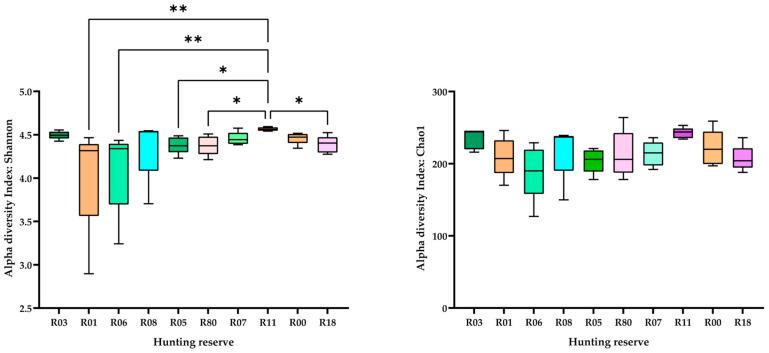
Alpha diversity calculated with the Shannon and Chao1 indices and significant (FDR, *p* < 0.05) pairwise comparison of the fecal microbiota of red deer in the 10 hunting reserves (* = *p* < 0.05 and ** = *p* < 0.01).

Alpha diversity was calculated using the Shannon and Chao1 indices (Figure 4) and pairwise comparisons were reported for FDR less than 0.05. Whilst for the Chao1 index no significant differences were calculated between hunting reserves, the Shannon index showed that R11 differed from the other five hunting reserves (R01, R06, R05, R80 and R18).

**Figure 5:**
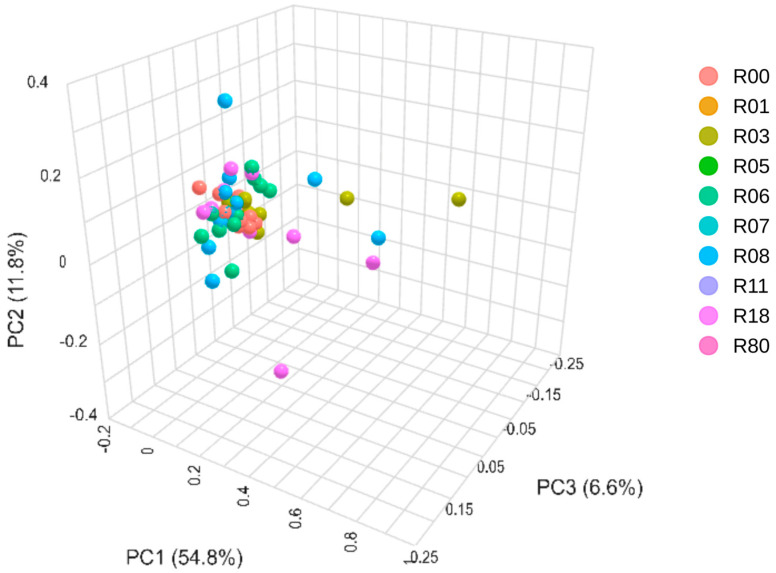
Principal coordinate analysis of Bray–Curtis beta diversity at a feature level of the fecal microbiota of red deer in the 10 hunting reserves.

The taxonomic composition of the microbial community measured in the feces at the taxonomic level was analyzed using the Bray–Curtis dissimilarity, an index of beta diversity (Figure 5). Although no significant difference was calculated after FDR correction for multiple testing (Table S1), R11 was most different from the other seven hunting reserves (R01, R06, R05, R07, R80, R00 and R18), followed by R07, which was significantly different from R80, R18 and R05, as shown for the Shannon alpha diversity index.

**Figure 6:**
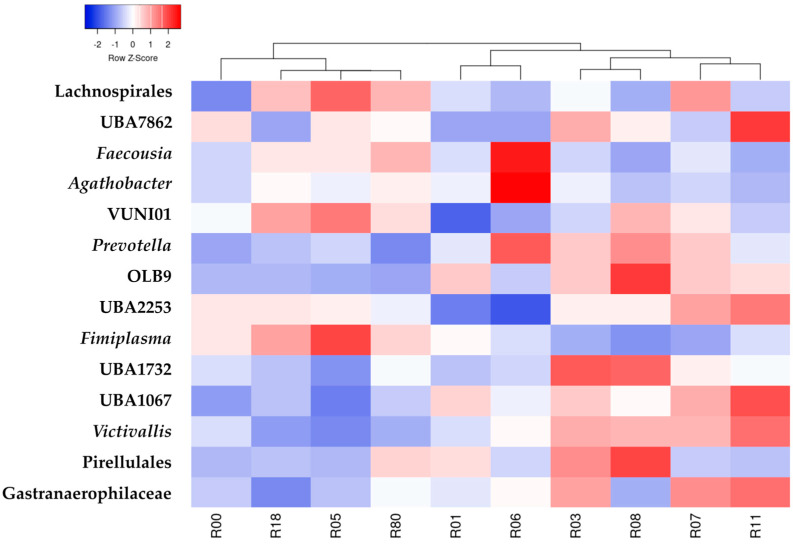
Cluster analysis of standardized linear discriminant analysis (LefSe) effect size scores applied to taxa that differed significantly between hunting reserves (LDA score > 2 and *p* < 0.01).

The comparison between hunting reserves of the microbiota in deer feces was tested using the LefSe method, and a description of the microbial population composition at the family and genus level in each hunting reserve can be found in Tables S2 and S3. Only taxa with a mean RA greater than 0.5% are reported. Of the top 10 abundant families, six belonged to the phylum Firmicutes (Lachnospiraceae, Oscillospiraceae_88309, CAG_272, Acutalibacteraceae, CAG_74 and Ruminococcaceae) and four to the phylum Bacteroidota, (Bacteroidaceae, UBA932 and Muribaculaceae). As shown in Table S3, of the 10 most abundant genera, *Faecousia*, RUG13077, PeH17, *Pradoshia*, *Avispirillum*, SFMI01, and CAG_83 belonged to the phylum Firmicutes and *Cryptobacteroides Paramuribaculum* and *Phocaeicola*_A_858004 to the phylum Bacteroidota. Cluster analysis of the reserves based on taxa that differed significantly in the LefSe analysis (*p* < 0.01) resulted in a first cluster with R03, R08, R07 and R11, a second cluster with R01 and R06, a third cluster with R18, R05 and R80 and an outgroup with R00. In the first cluster, *Faecousia*, *Agathobater* and *Fimiplasma* were underexpressed and, in contrast, *Prevotella* (with the exception of R11), OLB9, UBA7862, UBA2253, UBA1732, UBA1067, *Victivallis* and Gastranaerophilaceae (with the exception of R08) were overexpressed. For the third cluster (R05, R18 and R80), opposite expressions were found for these features. A distinct signature was observed for the second cluster (R01 and R06), with lower expression of Lachnospirales, UBA7862, UBA2253, VUNI01 and UBA1732 (Figure 6).

## 4. Discussion

This is the first study to describe the fecal bacterial microbiota of red deer in the context of different FVG hunting reserves, using high-throughput sequencing.

The composition of the fecal microbiota of red deer confirms that Firmicutes and Bacteroidota are the predominant phyla, as reported in previous studies [1,4,20,21,22] for different deer species. The Greengene Classifier 2024.09 (https://ftp.microbio.me/greengenes_release/current/ (accessed on 21 August 2025) categorizes Firmicutes into A, B, C and D, which are also referred to as Bacillota, although the renaming of the phylum Firmicutes is still controversial among microbiologists [23,24]. Bacteria of the phylum Firmicutes_A are mainly Clostridia in the gut and degrade fibers and glycans, producing short-chain fatty acids and energy for the host [25,26]. In addition, a higher ratio of Firmicutes to Bacteroidota indicates a higher efficiency of fermentation [20,27]. Wang et al. [22] observed that Firmicutes and the ratio of Firmicutes to Bacteroidota were higher in geographical locations with lower temperatures, and You et al. [21] showed a variation of these phyla also depending on the season and the type and availability of food resources. The ratio of Firmicutes to Bacteroidota decreased from 3.32 in wild deer to 2.56 in winter-reared deer and 1.88 in year-round reared deer, although the authors did not report whether the differences were significant [1]. The fecal microbiota of wild alpine musk deer (*Moschus chrysogaster*) was richer in Firmicutes than in captive animals [20]. Further differences in microbiota composition were observed in the relative abundance of Patescibacteria and Proteobacteria, which was very low compared to that reported by [21,22]. The latter authors showed that Proteobacteria had almost disappeared in the grassy season compared to the withering season. The relative abundance of Protebacteria in captive alpine musk deer was much higher than in wild animals [20]. In this study, the diet was largely different: leaves and branches in the wild animals and carrots, corn and forage in the captive deer. Conversely, Víquez-R et al. [1] reported a higher abundance of Proteobacteria in free-ranging deer compared to animal winter gated or all year-round gated.

Based on the literature, one would have expected a higher RA of Firmicutes and a higher ratio of Firmicutes to Bacteroidota. The reason why R11 has a lower abundance of Firmicutes_A and Firmicutes overall and a lower ratio of Firmicutes_A to Bacteroidota compared to R18 and R80 is not easy to explain. Part of the differences may be related to the hypervariable regions and the classifier used for annotation. Other factors such as climatic conditions, precipitation and water resources as well as slope could be involved in the observed differences.

However, a comparison of the alpha diversity indices offers a different perspective. The Chao1 index refers to richness and the Shannon index to the richness and abundance of taxa. The higher value of the latter index in R11 and the lower one in R01 and R06 indicates that both richness and diversity were influenced by the sampling area (Figure 1; Table 1). R11 is the reserve in the Tarvisio forest in the extreme east of the FVG region, while the R06 and R01 reserves are in the extreme west. The R11 habitat is mainly occupied by fir, spruce and beech forests, while R06 also hosts heathlands and shrublands and R01 includes *Ostrya carpinifolia* forests and anthropogenic grasslands. The different composition and availability of trophic resources may have influenced fecal microbiota. However, not only the vegetation but also the climatic conditions and the location of the reserves could have influenced the variability of the microbiota. R11, R03, R08 and R07 are located in the mountains, R01 and R80 are close to the lowlands and anthropogenic areas and R06 has rocky soil and is the only reserve with extensive heathland and shrublands. Looking at the composition of the fecal microbiota, it is likely that the higher biodiversity observed in the deer populations of the mountainous regions gives the deer more resilience and robustness when living under adverse conditions [28,29,30].

The variation in beta diversity, which nearly mirrors the Shannon diversity index, suggests that the population of bacteria inhabiting the deer gut also varies. Deer feces sampled in the batches of the mountain (R03, R08, R11 and R07) were characterized by bacteria with high fiber degradation capacity, not necessarily belonging to the phylum Firmicutes. *Prevotella*, phylum Bacteroidota, possessed ologisaccharolytic and xylanolytic activity [31], and Christensenellaceae also efficiently degrade cellulose to acetic and butyric acid [32]. In addition, *Victivallis* is a strictly anaerobic Gram-negative bacterium that degrades cellobiose and has been found in human feces [33] and is involved in rumen metabolism to reduce urinary N excretion [34]. Victivallales are involved in the production of extracellular mucus in the intestine and may contribute to the improvement of intestinal function [35]. Interestingly, the genera of this order were underexpressed in the reserves of the third cluster (R18, R05 and R80) and R00. The modulation of *Faecousia*, phylum Firmicutes_A and family Oscillospiraceae, may be associated with coevolution with the host to modulate energy metabolism, as hypothesized in highland deer compared to plateau deer [36]. The functions of *Agathobacter* in deer and ruminants have not yet been reported, but in humans it is involved in fiber utilization [37]. Although *Faecousia* and *Agathobacter* may be involved in fiber degradation, it is likely that they were replaced by other fiber-fermenting bacteria that occupied a different ecological niche than the deer that remained in the R06 or R80 reserves, which were characterized by different climatic and trophic conditions.

Although some biologically meaningful results were obtained, this study has some limitations due to the analysis of wild animal samples. Limiting factors include the sex, age and health status of the animals. However, there are few studies examining the effects of age and sex, and some authors reported little or no difference in gut microbiota [10,38,39]. In addition to feed resources, competition with other herbivores and environmental variables may also influence the composition of the gut microbial population.

These are preliminary results of gut microbiota variations obtained in a limited number of wild red deer, which may be interesting to study the adaptation of wildlife to different habitats in a small area characterized by strong biological, ecological and landscape differences.

## 5. Conclusions

The results demonstrate that the fecal microbiome of red deer is influenced by the sampling site, probably due to dietary variation in different habitats and vegetation. The variation in fecal microbiota may depend on the type of fiber available in the hunting reserves, with higher diversity and resilience of the microbial population in the mountainous areas than in the hills and lowlands. The collected results underline the paramount role of the microbiota in the nutritional ecology of red deer and can be considered as baseline data for the study of their relationship with the environment. Nevertheless, the shifts in the microbiota deserve further studies to investigate the mechanisms of these interactions.

## Figures and Tables

**Table 1:** Main characteristics of the hunting reserves in which the fecal samples of deer were collected [15].

Code	Hunting Reserve Name	Rainfall, mm/y	Altitude (a.s.l.), m	Average T, °C	Vegetation
R00	Tramonti	1600–2400	448–1558	0–12	Fir woods
R01	Aviano	1500–2500	168–414	0–14	*Ostrya carpinifolia* forests, fir woods, anthropogenic grasslands
R03	Arta Terme	1400–2300	993–1318	0–12	Beech forest, alpine and subalpine grasslands
R05	Gemona del Friuli	1500–2300	619–1175	0–14	Beech forest, *Quercus pubescens* and *Ostrya carpinifolia* forests, anthropogenic grasslands
R06	Erto e Casso	1600–2400	711–853	0–12	Fir woods, spruce forests and beech forests heathlands, shrublands
R07	Paularo	1500–3400	665–1373	0–12	Fir woods, spruce forests and beech forests
R08	Forni Avoltri	1400–2300	993–1198	0–12	Fir woods, spruce forests, alpine and subalpine grasslands, heathlands, shrublands
R11	Tarvisio-Malborghetto	1500–3400	1070–1420	0–12	Fir woods, spruce forests and beech forests
R18	Trasaghis	1500–2300	182–427	0–14	Beech forest, *Quercus pubescens* and *Ostrya carpinifolia*, *Pinus nigra*, *Pinus silvestris forests*, riparian habitats
R80	Meduno	1500–2500	299–1009	0–14	*Ostrya carpinifolia* forests, fir woods, anthropogenic grasslands

## Data Availability

The datasets generated for this study can be found at NCBI/BioProject/PRJNA1291720 (https://www.ncbi.nlm.nih.gov/bioproject/ PRJNA1291720 (accessed on 15 July 2025)).

## Supplementary Materials

The following supporting information can be downloaded at: https://www.mdpi.com/article/10.3390/ani15172517/s1, Table S1: Pairwise comparisons (*p* < 0.05) of beta diversity index measured in the feces of red deer samples in the 10 hunting reserves; Table S2: Family of fecal microbiota of red deer with a mean Relative abundance (%) higher than 0.5% observed in the 10 hunting reserves; Table S3: Genera of fecal microbiota of red deer with a mean Relative abundance (%) higher than 0.5% observed in the 10 hunting reserves.

## Abbreviations

The following abbreviations are used in this manuscript:

RA	Relative abundance
